# Nasopharyngeal carriage of *Streptococcus pneumoniae*, *Haemophilus influenzae*, and *Staphylococcus aureus* in a Brazilian elderly cohort

**DOI:** 10.1371/journal.pone.0221525

**Published:** 2019-08-22

**Authors:** Rosemeire Cobo Zanella, Maria Cristina de Cunto Brandileone, Samanta Cristine Grassi Almeida, Ana Paula Silva de Lemos, Claudio Tavares Sacchi, Claudia R. Gonçalves, Maria Gisele Gonçalves, Lucila Okuyama Fukasawa, Marcos Daniel Saraiva, Luís Fernando Rangel, Julia Lusis Lassance Cunha, Thereza Cristina Ariza Rotta, Christian Douradinho, Wilson Jacob-Filho, Ruth Minamisava, Ana Lúcia Andrade

**Affiliations:** 1 National Laboratory for Meningitis and Pneumococcal Infections, Centre of Bacteriology, Institute Adolfo Lutz (IAL), São Paulo, Brazil; 2 Strategic Laboratory, Institute Adolfo Lutz (IAL), São Paulo, Brazil; 3 Molecular Biology, Centre of Immunology, Institute Adolfo Lutz (IAL), São Paulo, Brazil; 4 Medical Research Laboratory in Aging (LIM 66) of Geriatrics Division of the Clinics Hospital of the University of São Paulo Medical School (GDCH-USPMS), São Paulo, Brazil; 5 School of Nursing, Federal University of Goiás, Goiânia, Brazil; 6 Institute of Tropical Pathology and Public Health, Federal University of Goiás, Goiânia, Brazil; Universidade de Lisboa Faculdade de Medicina, PORTUGAL

## Abstract

We aimed to investigate the nasopharyngeal colonization (NPC) by *Streptococcus pneumoniae*, *Haemophilus influenzae*, and *Staphylococcus aureus* in the elderly population and to assess the demographic factors associated with NPC. This was an observational cohort study in which outpatients aged ≥60 years were enrolled from April to August 2017, with a follow-up visit from September through December 2017. Nasopharyngeal (NP) swabs were collected, bacteria were detected and isolated, and isolates were subjected to phenotypic and molecular characterization using standard microbiological techniques. At enrolment, the rates of *S*. *aureus*, methicillin-resistant *S*. *aureus* (MRSA), *H*. *influenzae*, and *S*. *pneumoniae* among 776 elderly outpatients were 15.9%, 2.3%, 2.5%, and 2.2%, respectively. Toxin production was detected in 21.1% of methicillin-susceptible *S*. *aureus*, and three SCC*mec* types were identified: II/IIb, IVa, and VI. At the follow-up visit, all carriage rates were similar (p > 0.05) to the rates at enrolment. Most of *S*. *pneumoniae* serotypes were not included in pneumococcal conjugate vaccines (PCVs), except for 7F, 3, and 19A. All strains of *H*. *influenzae* were non-typeable. Previous use of antibiotics and 23-valent pneumococcal polysaccharide vaccination (p < 0.05) were risk factors for *S*. *aureus* and MRSA carriage; *S*. *aureus* colonization was also associated with chronic kidney disease (p = 0.021). *S*. *pneumoniae* carriage was associated with male gender (p = 0.032) and an absence of diabetes (p = 0.034), while not receiving an influenza vaccine (p = 0.049) and chronic obstructive pulmonary disease (p = 0.031) were risk factors for *H*. *influenzae* colonization. The frailty of study participants was not associated with colonization status. We found a higher *S*. *aureus* carriage rate compared with the *S*. *pneumoniae*- and *H*. *influenzae*-carriage rates in a well-attended population in a geriatric outpatient clinic. This is one of the few studies conducted in Brazil that can support future colonization studies among elderly individuals.

## Introduction

The global elderly population is growing significantly each year, leading to a high risk for community-acquired pneumonia (CAP) and, consequently, higher mortality due to CAP [1]. In Europe, a comprehensive review analyzed 46 studies on CAP in adults and elderly patients. Isolates were retrieved from different clinical specimens (blood, pleural fluid, bronchial lavage, urine, pleural fluid, and sputum), and *Streptococcus pneumoniae* was the most frequent cause of CAP (35%), followed by *Haemophilus influenzae* (13%) and *Staphylococcus aureus* (<7%) [2]. *S*. *pneumoniae* and *S*. *aureus* have been identified in approximately 2.5% of elderly patients with post-influenza bacterial pneumonia [3]. *S*. *aureus* and methicillin-resistant *S*. *aureus* (MRSA) are associated with exposure to healthcare facilities [4,5].

Bacterial colonization of the upper respiratory tract is asymptomatic and represents the first step of CAP establishment in the host. Therefore, sampling of nasopharynx secretions has been largely used to understand the circulation of respiratory bacteria [6]. Studies on nasopharyngeal colonization (NPC) by *S*. *pneumoniae* and *H*. *influenzae* have been largely conducted in young children and have provided valuable information on the interaction between carriage and disease. Co-colonization studies related to *S*. *pneumoniae*, *H*. *influenzae*, and *S*. *aureus* have shown that *S*. *pneumoniae* and *H*. *influenzae* exhibit synergistic nasopharyngeal (NP) co-habitation, whereas *S*. *aureus* tends to show a negative association with *S*. *pneumoniae* and *H*. *influenzae* [7,8].

Nevertheless, there is limited data regarding bacterial NPC among elderly adults worldwide [9]. No study has evaluated the carrier status in the elderly population in Brazil, which would be extremely important for guiding antimicrobial therapy and implementing preventive measures such as immunization [10]. In Brazil, the National Immunization Program of the Ministry of Health provides the following vaccines for individuals aged ≥ 60 years: influenza, hepatitis B, and DT (diphtheria and tetanus). The 23-valent pneumococcal polysaccharide (PPSV23) is recommended only on medical advice [11]. The aim of this study was to estimate the carriage rates of *S*. *pneumoniae*, *H*. *influenzae*, and *S*. *aureus* in asymptomatic elderly outpatients living in São Paulo, Brazil. We also assessed demographic and other potential factors influencing NPC.

## Material and methods

### Study design and population

This was an observational study assessing a cohort of outpatients attending the Geriatrics Division of the Clinics Hospital of the University of São Paulo Medical School, a public tertiary referral hospital located in the São Paulo municipality, that attends to about 1,400 patients annually. The municipality of São Paulo is the largest city in Brazil, with about 12 million inhabitants. Subjects were enrolled from April through August 2017 (fall-winter, visit 1). The follow-up visit was scheduled from September through December 2017 (spring-summer, visit 2). Eligible participants were aged 60 years or more. The exclusion criteria at the time of the interviews (visit 1 and follow-up visit) were clinical manifestations of infectious diseases, hospitalization, and antibiotic treatment within 7 days prior to data collection.

#### Data collection

Baseline demographic information, clinical presentation, and hospital course were recorded in a web-based secure electronic case report form [12]. The clinical and epidemiological characteristics of the participants that were assessed included gender, living in a long-term institution, living with a child (0–18 years old), prior exposure to 23-valent pneumococcal polysaccharide (PPSV23) and influenza vaccines, race/ethnicity, marital status, education level, hospitalization in the last 6 months, antibiotic use in the last 12 months, and frailty-evaluated by the Study of Osteoporotic Fractures (SOF) Frailty Scale [13]. Co-morbidity information related to diabetes, chronic obstructive pulmonary disease (COPD), chronic kidney disease, asthma, and smoking status was collected from medical charts. All enrolled individuals were invited to participate in the follow-up visit. Epidemiologic data including those pertaining to demographic and risk factors were collected by trained field staff. The same study methodology and inclusion/exclusion criteria for enrolment were used in both visits. The sample size for visit 1 (~700 individuals) was calculated based on an estimated 3% colonization rate by *S*. *pneumoniae* in the elderly population (2% error, 95% confidence interval [CI], and an effect design of 2.0), considering that an additional 20% of individuals would likely not meet the eligibility criteria [14].

#### Ethics statement

The study was approved by the Ethics Committee of the University of São Paulo Medical School, São Paulo, Brazil (No 1794653). All samples and questionnaires were coded after collection and processed in the laboratory using the same identification code for both visits. Data were stored in a dedicated database for heightened data security.

### Sample collection and laboratory procedures

One single NP swab was collected and stored according to the World Health Organization working group standard methods using flexible flocked sterile swabs. Swabs were placed into 1.2 mL of STGG (skim milk, tryptone, glucose, and glycerin) liquid transport medium, stored in a cool box, and transported to the Center of Bacteriology at Adolfo Lutz Institute within 3–4 hours of collection [15]. Samples were then vortexed and stored at -70°C. For culture, 120 μL of the thawed STGG was inoculated into TYS broth (Tood-Hewitt broth supplemented with yeast extract and rabbit serum), for enrichment culture; after 6 hours of incubation at 35 ± 2°C in 5% CO_2_, 10 μL of TYS was inoculated on a sheep blood agar plate. Another STGG aliquot (120 μL) was inoculated on chocolate agar supplemented with IsoVitalex and 300 mg/L bacitracin, and one more aliquot (100 μL) was inoculated on mannitol salt agar (MSA). The blood agar and chocolate plates were incubated at 35 ± 2°C in 5% CO_2_, and the MSA plate was incubated at 35 ± 2°C for 48 h. The plates were screened for suspect colonies (1 to 3 colonies) of *S*. *pneumoniae*, *H*. *influenzae*, and *S*. *aureus*. Pneumococcal identification was based on alpha-hemolysis on the blood agar plate and optochin susceptibility and bile solubility tests. *S*. *pneumoniae* was serotyped by Quellung reaction with antisera from Statens Serum Institut (Copenhagen, Denmark). Quellung non-typeable pneumococcus strains were tested in eight sequential conventional PCR multiplex assays, which covered 70 serotypes plus the capsule (*cps*) gene [16]. Penicillin (Pen) and ceftriaxone (Ctx) susceptibility was assessed using an antimicrobial-susceptibility strip test (Liofilchem, Italy), following the CLSI recommendations [17]; the resistance breakpoints for the minimum inhibitory concentration testing (MIC) were ≥0.12 mg/L and ≥1.0 mg/L for Pen and Ctx, respectively. *H*. *influenzae* was confirmed using the *hpd*3 target and genotyped by a qPCR assay [18, 19]. *S*. *aureus* screening was performed by Staphclin Latex (Laborclin, Brazil); species confirmation (*nuc* gene), detection of oxacillin resistance (*mec*A gene), and evaluation of toxin production (Panton-Valentine leukocidin—PVL, toxic shock syndrome toxin 1 -TSST-1, and staphylococcal enterotoxins—SE types A-E) were performed by qPCR [20–22]. Multiplex PCR was used to characterize staphylococcal cassette chromosome *mec* (SCC*mec*) elements [23].

*S*. *pneumoniae* and *H*. *influenzae* were also detected by qPCR. An STGG aliquot (500 μl) was incubated with lysozyme (0.015 g/mL) and mutanolysin (25 U/mL) (Sigma Chemicals) [24]. DNA was extracted and purified using the Purelink^™^ Genomic DNA Mini Kit (Invitrogen) and concentrated 5-fold with elution buffer. DNA was tested by qPCR for the presence of the *lytA* gene (*S*. *pneumoniae*), *hpd3* gene (*H*. *influenzae*), and human *RNaseP* gene [18]. Multiplex qPCR serotype assay for 21 serotypes was performed on all *lyt*A-positive NP specimens [16]. Samples with positive qPCR results for *S*. *pneumoniae* (Ct ≤ 30) but with negative results in the multiplex qPCR serotype assay were analyzed by conventional multiplex PCR to detect other possible serotypes [24]. *H*. *influenzae*-positive samples were genotyped in qPCR assays to detect six serotypes [19].

### Data analysis

The primary outcomes were colonization by *S*. *pneumoniae* and *H*. *influenzae* (culture and qPCR) and *S*. *aureus* (culture). Co-colonization was defined when the participant was simultaneously colonized by ≥2 bacterial species. We investigated the rates and respective 95% confidence intervals of *S*. *pneumoniae*, *S*. *aureus*, and *H*. *influenzae* colonization for participants attending both visit 1 and the follow-up visit. At enrolment, we compared demographics and clinical characteristics between the carrier and non-carrier groups of *S*. *aureus*, MRSA, *S*. *pneumoniae*, and *H*. *influenzae*. A descriptive analysis was performed with the clinical and demographic characteristics of the participants. The Mann-Whitney test was used to test for differences in medians of age between men and women. The chi-squared or Fisher’s exact test were used to compare the proportions of variables between the carrier and non-carrier groups. P-values < 0.05 were considered statistically significant.

## Results

A total of 820 participants were recruited in visit 1, of which 44 (5.1%) were excluded due to antibiotic use in the last 7 days (n = 26; 3.2%), refusal to participate in the study (n = 7; 0.9%), inability to speak Portuguese (n = 1; 0.1%), clinical decompensation (n = 2; 0.2%), cognitive impairment severe enough to compromise understanding of the NP swab (n = 4; 0.5%), sample collection performed in duplicate (n = 2; 0.2%), and intercurrence during sample transport (n = 2; 0.2%). Thus, 776 elderly subjects were included in assessments at visit 1 (mean age: 81.5 years; range: 60–102 years). Of these, 192 (22.3%) were lost to follow-up due to non-attendance (n = 148; 19.1%), death (n = 25; 3.2%), antibiotic use in the last 7 days (n = 16; 2.7%), and refusal to participate (n = 3; 0.5%). Thus, 584 (75.3%) participants were included for analysis at the follow-up visit and their mean age was 81.9 years (range: 60–103 years old). The mean interval between visit 1 and the follow-up visit was 133 days.

Demographic and clinical characteristics of the participants during visit 1 and the follow-up visit are shown in Table 1. Overall, females (mean, 82 yr; range, 60–102 yr) were older than males (mean, 80.5 yr; range, 61–96 yr) (p = 0.005). About 70% of the participants were female, and only 2% lived in a long-term institution. Most participants reported no COPD, no asthma, and no smoking and had received the influenza vaccine in 2017 (88.92%; 690/776).

**Table 1:** Demographic and clinical characteristics of elderly participants in both visit 1 and the follow-up visit (visit 2)^a^.

Characteristics	Visit 1 n = 776		Visit 2 n = 584	
	N	%	n	%
Female	554	71.39	429	73.46
Living in a long-term institution	13	1.68	10	1.71
Living with a child	134	17.27	108	18.49
no information	1	0.13	1	0.17
Influenza vaccine	690	88.92	538	92.12
PPSV23 vaccine^b^	203	26.16	180	30.82
Caucasian	442	56.96	330	56.51
Marriage	264	34.02	200	34.25
School education (≤4 years)	563	72.55	425	72.77
Hospitalization in the last 6 months	117	15.08	76	13.01
Antibiotic use in the last 12 months	338	43.56	252	43.15
Diabetes				
Yes	281	36.21	208	35.62
No	495	63.79	376	64.38
COPD^c^				
Yes	51	6.57	35	5.99
No	725	93.43	549	94.01
Asthma				
Yes	35	4.51	29	4.97
No	741	95.49	555	95.03
Chronic Kidney Disease				
Yes	172	22.16	127	21.75
No	604	77.84	457	78.25
Smoking				
Yes	100	12.89	75	12.84
No	676	87.11	509	87.16
SOF Frailty Scale^d^				
Robust	282	36.34	210	35.96
Pre-frail	275	35.44	209	35.79
Frail	219	28.22	165	28.25

^a^ The median age was 81.5 years (range: 60–102 years) for visit 1 and 81.9 years (range: 60–103 years) for the follow-up visit.

^b^ PPSV23 vaccine, 23-valent pneumococcal polysaccharide vaccine

^c^ COPD, chronic obstructive pulmonary disease

^d^ SOF, Study of Osteoporotic Fractures

NP-carriage data are provided in Table 2. Similar colonization rates were found during visit 1 and in the follow-up visit for all three bacteria. In visit 1, *S*. *aureus* was found in 15.9% (123/776) of the participants, and 2.3% (18/776) harbored MRSA. Three distinct SCC*mec* types were characterized among the MRSA strains (7 II/IIb, 5 IVa, 2 VI), while in four SCCmec type could not determine (ND). Toxin and SE genes were detected in 21.1% (26/123) of the *S*. *aureus* strains; 17 *S*. *aureus* strains harbored one unique gene (5 TSST-1, 4 SEA, 3 SEB, 2 SEC, 2 PVL, and 1 SEA), whereas multiple virulence genes were identified in 9 *S*. *aureus* strains (4 TSST-1/SEA, 3 SEA/SEB, 1 PVL/SEA/SEB, and 1 SEA/SEC). All 26 toxin-producing *S*. *aureus* strains were methicillin-sensitive (MSSA). Among all *S*. *aureus* carriers, 9.1% (n = 53) showed colonization in both visits. Data on the MRSA-carriage characteristics and the respective types of SCC*mec* elements is given in the supplemental material S1 Table.

**Table 2:** Nasopharyngeal carriage prevalence of *Streptococcus pneumoniae*, *Staphylococcus aureus*, methicillin-resistant *Staphylococcus aureus* (MRSA), and *Haemophilus influenzae* in elderly individuals > 60 years of age considering the subset of participants seen in both visit 1 and the follow-up visit (visit 2; n = 584).

Carriage	n = 776			n = 584^a^					
	Visit 1			Visit 1			Visit 2		
	n	%	95% CI	n	%	95% CI	n	%	95% IC
*S*. *aureus*	123	15.9	13.5–18.6	91	15.6	12.9–18.8	105	18.0	15.1–21.3
MRSA	18	2.3	1.5–3.6	12	2.1	1.2–3.6	18	3.1	2.0–4.8
*S*. *pneumoniae*	17	2.2	1.4–3.5	9	1.5	0.8–2.9	15	2.6	1.6–4.2
*H*. *influenzae*	19^b^	2.5	1.6–3.8	14^c^	2.4	1.4–4.0	10^d^	1.7	0.9–3.1

^a^ Subset composed of the same individuals for visit 1 and follow-up visit (visit 2)

^b,c,d^ All non-typeable isolates

At visit 1, *S*. *pneumoniae* was detected in 2.2% (17/776) of the participants, with the following non-preventable serotypes/genotypes (6C, 35B, 20, 15B, 23A, 22F/22A, one each; 34, 9N, 28A, two each); in five pneumococcal qPCR-positive samples, the serotype/genotype was not determined (ND). In the follow-up visit (2.6%, 15/584; the serotypes/genotypes were 3, 7F, 13, 15C, 28A, 34, [one each]; 19A, 23A [two each], and five not determined [ND] pneumococcal qPCR-positive samples), only three preventable serotype/genotypes (3, 7F, and 19A) were detected. Among the 9 *S*. *pneumoniae* carriers identified in both visit 1 and the follow-up visit, only one showed the same serotype 34. Three *S*. *pneumoniae* strains were considered Pen-resistant, two with MIC = 0.125 mg/L, one in visit 1 (type 6C) and one in the follow-up visit (type 15C), and one with MIC = 2 mg/L (type 19A) that was also Ctx-resistant (MIC = 1 mg/L) in the follow-up visit. *H*. *influenzae* was found in 2.5% (19/776) of the samples in visit 1 and 1.7% (10/584) in the follow-up visit. Among the 14 *H*. *influenzae* carriers, only one showed colonization in both visits. qPCR enabled detection of six pneumococcal carriers at visit 1 and another six cases at the follow-up visit that were negative by culture, and 7 and 4 cases of *H*. *influenzae* in the respective visits with negative cultures. The data are presented in the supplemental material (S2 Table).

Co-colonization was identified among 9 participants (1.1%) at visit 1: *S*. *pneumoniae—H*. *influenzae—S*. *aureus* and *S*. *pneumoniae—H*. *influenzae* (n = 1 each), *H*. *influenzae—S*. *aureus* (n = 3, one MRSA), and *S*. *pneumoniae—S*. *aureus* (n = 4). At visit 2, five other participants were carrying *S*. *pneumoniae—S*. *aureus* (n = 3), *H*. *influenzae—S*. *pneumoniae* (n = 1), or *H*. *influenzae—S*. *aureus* (n = 1).

We did not detect differences in age between males and females in carriage, for any of the studied bacteria (Mann-Whitney test, p > 0.05). The risk factors associated with *S*. *aureus* and MRSA colonization were the use of antibiotics in the last 12 months and PPSV23 vaccination (p < 0.05); *S*. *aureus* was also associated with chronic kidney disease (p = 0.021). *S*. *pneumoniae* carriage was associated with male gender (p = 0.032) and an absence of diabetes (p = 0.034); and failure to receive an influenza vaccine (p = 0.049). COPD (p = 0.031) was associated with *H*. *influenzae* carriage. Frailty was not associated with carriage by *S*. *aureus*, *S*. *pneumoniae*, and *H*. *influenzae* (Table 3).

**Table 3:** Demographic characteristics, medical conditions, and risk factors for carriage of *Staphylococcus aureus*, methicillin-resistant *Staphylococcus aureus* (MRSA), *Streptococcus pneumoniae*, and *Haemophilus influenzae* in visit 1 among elderly participants^a^ (N = 776).

Variable	*S*. *aureus*			MRSA			*S*. *pneumoniae*			*H*. *influenzae*				Total
	positive	%	p-value	positive	%	p-value	positive	%	p-value	Positive	%		p-value	
Gender														
Female (n = 554)	80	14.4	0.089	11	2.0	0.329	8	1.4	0.032	12	2.2	0.421		554
Male (n = 222)	43	19.4		7	3.2		9	4.1		7	3.2			222
Living in a long-term institution														
No (n = 763)	120	15.7	0.444	17	2.2	0.265	16	2.1	0.252	19	2.5	-		763
Yes (n = 13)	3	23.1		1	7.7		1	7.7		0	0.0			13
Living with child														
No (n = 642)	101	15.8	0.849	18	2.8	-	15	2.3	0.751	15	2.3	0.757		641
Yes (n = 134)	22	16.4		0	0.0		2	1.5		4	3.0			134
Influenza vaccine														
No (n = 86)	11	12.8	0.410	0	0.0	-	3	3.5	0.422	5	5.8	0.049		86
Yes (n = 690)	112	16.2		18	2.6		14	2.0		14	2.0			690
PPSV23 vaccine^b^														
No (n = 573)	82	14.3	0.048	8	1.4	0.011	13	2.3	1.000	13	2.3	0.600		573
Yes (n = 203)	41	20.2		10	4.9		4	2.0		6	3.0			203
Marriage														
No (n = 512)	79	15.4	0.655	13	2.5	0.572	9	1.8	0.251	12	2.3	0.793		512
Yes (n = 264)	44	16.7		5	1.9		8	3.0		7	2.7			264
School education														
≤4 years (n = 563)	89	15.8	0.958	12	2.1	0.596	13	2.3	1.000	16	2.8	0.249		563
≥5 years (n = 213)	34	16.0		6	2.8		4	1.9		3	1.4			213
Hospitalization in previous 6 months														
No (n = 659)	104	15.8	0.901	14	2.1	0.333	15	2.3	1.000	16	2.4	1.000		659
Yes (n = 117)	19	16.2		4	3.4		2	1.7		3	2.6			117
Antibiotic use in the last 12 months														
No (n = 438)	59	13.5	.039	5	1.1	0.013	10	2.3	0.841	12	2.7	0.550		438
Yes (n = 338)	64	18.9		13	3.8		7	2.1		7	2.1			338
Diabetes														
No (n = 495)	81	16.4	0.603	12	2.4	0.797	15	3.0	0.034	13	2.6	0.671		495
Yes (n = 281)	42	14.9		6	2.1		2	0.7		6	2.1			281
COPD^c^														
No (n = 725)	113	15.6	0.447	17	2.3	1.000	14	1.9	0.095	15	2.1	0.031		725
Yes (n = 51)	10	19.6		1	2.0		3	5.9		4	7.8			51
Asthma														
No (n = 741)	116	15.7	0.492	18	2.4	-	15	2.0	0.176	19	2.6	-		741
Yes (n = 35)	7	20.0		0	0.0		2	5.7		0	0.0			35
Chronic kidney disease														
No (n = 604)	86	14.2	0.021	11	1.8	0.091	13	2.2	1.000	17	2.8	0.274		604
Yes (n = 172)	37	21.5		7	4.1		4	2.3		2	1.2			172
Smoking														
Yes (n = 676)	101	14.9	0.071	14	2.1	0.273	12	1.8	0.056	16	2.4	0.725		676
No (n = 100)	22	22.0		4	4.0		5	5.0		3	3.0			100
SOF Frailty Scale^d^														
Robust (n = 282)	42	14.9	0.837	4	1.4	0.422	5	1.8	0.816	6	2.1	0.712		282
Pre-frail (n = 275)	46	16.7		8	2.9		7	2.5		6	2.2			275
Frail (n = 219)	35	15.9		6	2.7		5	2.3		7	3.2			219

^a^ Visit 1 mean age: 81.5 years (range: 60–102 years old)

^b^ PPSV23 vaccine, 23-valent pneumococcal polysaccharide vaccine

^c^COPD, chronic obstructive pulmonary disease

^d^SOF Frailty Scale, Study of Osteoporotic Fractures

## Discussion

We examined NP carriage for three bacterial pathogens in elderly outpatients attending the biggest geriatric clinic of São Paulo city. The main finding of our study was the high prevalence of *S*. *aureus* carriers in contrast to the low prevalence of *H*. *influenzae* and *S*. *pneumoniae* carriers. These results are consistent with the reports on a co-habitation relationship of these three bacteria in children that show a synergic association between *H*. *influenzae* and *S*. *pneumoniae* in the respiratory mucosa ad the competition between these bacteria and *S*. *aureus* for the niche of the nasopharynx [25–27].

High rates of *S*. *aureus* in nasal swabs among adults ≥ 60 years old from community have been described in Brazil (17.8%), Australia (23.1%), Denmark (24.1%), UK (25.8%) and Germany (28.5%) [28–32]. Another Brazilian study that investigated the rate of nasal *S*. *aureus* carriers among adults from a primary-healthcare unit found a rate of 11.5% [33]. A Portuguese study on *S*. *aureus* prevalence that used NP swabs showed a 13.3% carriage rate [4], similar to the result found in our study. Little information is available on MRSA carriage in elderly individuals, with rates varying from 0.7 to 2.0% in Portugal, Brazil, Australian, Danish, and British studies, very similar to our findings (2.3%) [4,28–31]. In contrast, a higher MRSA colonization rate was found in a Maltese study (4.8%) among 83 healthy elderly, and in a recent Brazilian study (3.7%) among elderly people living in a nursing home, which is a setting with a higher risk for MRSA reservoirs and a source of dissemination to hospital environments [5,34]. None of the MRSA strains harbored virulence factor genes (i.e., PVL, TSST-1, and enterotoxins), similar to the results found in other studies [4,28,29]. However, we found that 20% of the MSSA isolates harbored these toxins and enterotoxins. A higher frequency of virulence genes in MSSA strains has been reported in studies with hospital strains [35,36]. This finding shows that despite the importance of knowledge about the dissemination of MRSA strains, knowledge of the spread of MSSA carrying virulence factors is also important for the health of the population, which also represents an important source of infection. Our results indicate that the previous use of antibiotics and chronic kidney disease were risk factors associated with colonization by *S*. *aureus* and MRSA. Among participants with chronic kidney disease, few were undergoing hemodialysis, so this specific risk factor cannot be assessed for this population. The MRSA carrier status was also associated with PPSV23 vaccination, which is an unexpected result since this vaccine does not act in the colonization state. Interactions among pathogens could be considered as a factor for this association, but co-colonization was found in only 1.0% of those vaccinated with PPV23, and 0.5% of the unvaccinated participants. Thus, the sample size was too small to support this possible interaction between *S*. *aureus* and *S*. *pneumoniae*.

Globally, a low NP prevalence of *S*. *pneumoniae* and *H*. *influenzae* has been reported in adults in contrast to the high rates usually found in children (~50%), although these pathogens are clinically relevant as a cause of CAP in children and elderly adults [10,37,38]. In Kenya, NP carriage of *S*. *pneumoniae* and *H*. *influenzae* in adults aged >50 years has been reported to be 4.7% and 2.8%, respectively [37]. In Finland, the NP prevalence of *S*. *pneumoniae* and *H*. *influenzae* was 5.3 and 1.0%, respectively [39]. Pneumococcal NP colonization has also been evaluated in adults >60 years in other countries, such as Belgium (5.5%), Israel (4.6%), and Italy (7.6%) [9,38,40]. It is noteworthy that in the present study, few participants lived with children (n = 134), and only 1.5% (n = 2) showed positive cultures for *S*. *pneumoniae* while 3% (n = 4) showed positive cultures for *H*. *influenzae*. As children are considered the main source for transmission of these bacteria in the community, this fact would partially explain the lower carriage rates. Although contact between elderly individuals and children is very common, residing with children is not so common for the elderly population.

We did not find differences in bacterial carriage rates between the fall-winter and spring-summer periods, although seasonality may influence NPC by respiratory pathogens. This finding may be due to the high rate of influenza vaccine immunization reported by the study participants. Studies worldwide have shown an association between *S*. *pneumoniae* colonization and diabetes [41], but our results did not show this association, being an unexpected result. Moreover, we found an association between pneumococcal colonization and male gender; although no differences in the distribution of gender and age of the study participants were observed.

In 2010, Brazil introduced the 10-valent pneumococcal conjugate vaccine (PCV10) as part of the childhood national immunization program; thus, this investigation was conducted in a scenario with high vaccine coverage [42]. As expected, despite the low number of strains of *S*. *pneumoniae* isolated, we observed that most serotypes were not contained in PCVs, except for serotypes 7F, 3, and 19A, as previously reported for IPD in elderly adults in Brazil [43]. Despite the low prevalence of *H*. *influenzae* in this study, all strains were NTHi, in line with the literature. After the introduction of Hib vaccination NTHi has become the most important type of *H*. *influenzae* not only in Brazil but also in several parts of the world [32,44]. Failure to receive influenza vaccine and to have COPD were risk factors associated with *H*. *influenzae* colonization, which may favor a higher occurrence of viral respiratory infections and thereby increase *H*. *influenzae* density in the nasopharynx of this population.

The demographic and clinical characteristics of the adult population as well as the potential risk factors and environmental factors associated with colonization also need to be considered when interpreting different findings on bacteria colonization between studies. We evaluated a very unique population that is generally not represented in colonization studies. The participants were elderly (mean age >80 years), approximately 60% being pre-frail or frail, most of them not living in a long-term institution, and showing a high rate of co-morbidities and antibiotic use over the last 12 months.

Limitations of our study should be mentioned. The ecological niches of the *S*. *aureus* strains are the anterior nares. We collected swabs from nasopharynx, which is not the primary niche for *S*. *aureus*. Even though we found a high rate of *S*. *aureus* colonization, the carrier rate in our study may still be underestimated. A multivariable logistic regression analysis would have been useful (where possible) to rule out possible confounding effects like the unbalanced distribution of age and sex. However, the number of isolates of *S*. *pneumoniae* and *H*. *influenzae* was too small, hampering accurate results in multivariable analysis due to the expected very wide confidence intervals. Another limitation would be that our study was restricted to the noninstitutionalized population (more than 98%), a fact that also contributed to the low MRSA carrier rate. In Brazil, traditionally the families take care of their elderly and institutionalization is still uncommon. In the State of São Paulo, about 0.8% of the elderly population resides in a long-term institution [45]. For MRSA strains, we only performed SCC*mec* typing which made it difficult to draw conclusions about the source of the acquisition (HA-MRSA or CA-MRSA lineages) of the isolates. Seventy percent of the participants were female, which can be considered as limitation of our study. We did not investigate oral samples (saliva or oropharyngeal), which could have improved *S*. *pneumoniae* and *H*. *influenzae* detection by qPCR as shown by others [46]. Different clinical specimens and diagnostic methods have been utilized for detection of *S*. *pneumoniae* and *H*. *influenzae* carriage among adult populations, making comparisons between studies difficult. Additional studies are of paramount importance to determine a better protocol for assessment of bacterial carriage in adults.

According to the Ministry of Health, in 2016, Brazil had the fifth largest elderly population worldwide. During 2012–2017, the Brazilian population aged ≥60 years grew by 18.8% [47]. Therefore, carriage studies involving elderly individuals are necessary for a better understanding of the colonization dynamics of *S*. *pneumoniae*, *H*. *influenzae*, and *S*. *aureus*, considering that colonization of the respiratory niche is the first step in disease development. In conclusion, we showed a high rate of *S*. *aureus* carriage compared with the low carriage rates for S. *pneumoniae* and H. *influenzae* in a well-attended population in a geriatric outpatient clinic. This is one of the few studies conducted in Brazil that could be used as the basis for future colonization studies in the elderly population.

## Supporting information

S1 Table Carriage patterns of the individuals colonized by *Staphylococcus aureus* or methicillin-resistant *Staphylococcus aureus* (MRSA) in this study. (DOCX)

S2 Table Carrier status considering the subset of participants seen in both visit 1 and the follow-up visit (visit 2; n = 584). (DOCX)
