# Supplementary material for: Nasopharyngeal carriage of Streptococcus pneumoniae, Haemophilus influenzae, and Staphylococcus aureus in a Brazilian elderly cohort
Source: PLoS One. 2019 Aug 22;14(8):e0221525. doi: 10.1371/journal.pone.0221525 (PMC6705818; doi:10.1371/journal.pone.0221525)
Supplement: S1 Table — (DOCX) [file pone.0221525.s001.docx]

**S1 Table**. Carriage characteristics of the individuals colonized by *Staphylococcus aureus* or methicillin-resistant *Staphylococcus aureus* (MRSA) in this study.

|  |  | **Visit** | | | | | | **Demographic characteristics** | | | | | | | | | |
| --- | --- | --- | --- | --- | --- | --- | --- | --- | --- | --- | --- | --- | --- | --- | --- | --- | --- |
| ID | Number of visits | Visit 1 (n=776) | | | Visit 2 (n=584 | | | Sex | Lives in a long-term institution | Living with child | Hospitalization in the last 6 months | Emergency department in the last 6 months | Difference in days between visits 1 and 2 | Use of antibiotics in the last 12 months | Charlson comorbidity index (CCI) | Years of schooling | Number of medicaments using at the time of collection |
|  |  | *S. aureus* | MRSA | SCC*mec type* | *S. aureus* | MRSA | SCC*mec type* |  |  |  |  |  |  |  |  |  |  |
| 280 | 1 | + | + | II | na | na | na | female | **-** | **-** | **-** | **+** | na | + | 5 | 4 | 9 |
| 20 | 1 | + | + | IVa | na | na | na | male | **-** | **-** | **-** | **+** | na | + | 4 | 11 | 11 |
| 411 | 1 | + | + | IVa | na | na | na | female | **-** | **-** | **-** | **-** | na | - | 3 | 14 | 6 |
| 432 | 1 | + | + | IVa | na | na | na | female | **-** | **-** | **-** | **+** | na | + | 5 | 5 | 13 |
| 486 | 1 | + | + | IVa | na | na | na | female | **-** | **-** | **-** | **-** | na | - | 5 | 4 | 9 |
| 143 | 1 | + | + | ND | na | na | na | male | **-** | **-** | **-** | **+** | na | + | 4 | 16 | 7 |
| 216 | 2 | + | + | II | + | + | VI | female | **-** | **-** | **-** | **+** | 119 | - | 7 | 4 | 11 |
| 231 | 2 | + | + | II | + | + | VI | female | **-** | **-** | **-** | **-** | 182 | + | 4 | 11 | 9 |
| 361 | 2 | + | + | II | + | + | IIb | male | **-** | **-** | **+** | **-** | 154 | + | 7 | 4 | 2 |
| 461 | 2 | + | + | IIb | + | + | IIb | female | **-** | **-** | **-** | **-** | 70 | + | 12 | 4 | 23 |
| 322 | 2 | + | + | IIb | - | na | na | female | **-** | **-** | **-** | **-** | 196 | + | 11 | 4 | 9 |
| 686 | 2 | + | + | IIb | - | na | na | male | **-** | **-** | **+** | **-** | 112 | + | 3 | 11 | 4 |
| 150 | 2 | + | + | IVa | - | na | na | female | **-** | **-** | **+** | **+** | 140 | + | 9 | 3 | 10 |
| 462 | 2 | + | + | VI | - | na | na | male | **+** | **-** | **-** | **+** | 175 | + | 10 | 4 | 14 |
| 666 | 2 | + | + | VI | - | na | na | male | **-** |  | **-** | **+** | 56 | - | 12 | 3 | 10 |
| 97 | 2 | + | + | ND | + | + | ND | female | **-** | **-** | **-** | **-** | 161 | + | 7 | 2 | 9 |

**Table S1 (cont)**. Carriage patterns of the individuals colonized by *Staphylococcus aureus* or methicillin-resistant *Staphylococcus aureus* (MRSA) in this study

|  |  | **Visit** | | | | | | **Demographic characteristics** | | | | | | | | | |
| --- | --- | --- | --- | --- | --- | --- | --- | --- | --- | --- | --- | --- | --- | --- | --- | --- | --- |
| ID | Number of visits | Visit 1 (n=776) | | | Visit 2 (n=584) | | | Sex | Lives in a long-term institution | Living with child | Hospitalization in the last 6 months | Emergency department in the last 6 months | Difference in days between visits 1 and 2 | Use of antibiotics in the last 12 months | Charlson comorbidity index (CCI) | Years of schooling | Number of medicaments using at the time of collection |
|  |  | *S. aureus* | MRSA | SCC*mec type* | *S. aureus* | MRSA | SCC*mec type* |  |  |  |  |  |  |  |  |  |  |
| 756 | 2 | + | + | ND | + | + | ND | male | - | - | - | - | 98 | - | 8 | 4 | 8 |
| 725 | 2 | + | + | ND | - | na | na | female | - | - | + | - | 140 | + | 5 | 1 | 8 |
| 304 | 2 | + | - | na | + | + | IIb | female | - | - | - | - | 190 | - | 9 | 4 | 16 |
| 611 | 2 | + | - | na | + | + | IIb | female | - | - | + | - | 70 | + | 8 | 8 | 10 |
| 424 | 2 | - | - | na | + | + | IIb | female | - | - | - | - | 168 | - | 6 | 2 | 10 |
| 525 | 2 | - | - | na | + | + | IIb | female | - | - | - | + | 91 | - | 7 | 4 | 9 |
| 258 | 2 | - | - | na | + | + | IVa | male | - | - | - | - | 112 | - | 9 | 1 | 4 |
| 335 | 2 | - | - | na | + | + | IVa | female | - | - | - | - | 133 | + | 5 | 0 | 9 |
| 209 | 2 | - | - | na | + | + | VI | female | - | - | - | - | 196 | - | 7 | 4 | 11 |
| 354 | 2 | - | - | na | + | + | VIII | male | - | - | + | - | 133 | + | 6 | 1 | 11 |
| 556 | 2 | - | - | na | + | + | IVa | female | - | - | + | + | 105 | - | 6 | 3 | 14 |
| 164 | 2 | - | - | na | + | + | ND | female | + | - | - | + | 112 | + | 4 | 11 | 12 |
| 425 | 2 | - | - | na | + | + | ND | male | - | - | - | - | 77 | - | 7 | 5 | 12 |
| 652 | 2 | - | - | na | + | + | ND | female | - | - | - | + | 154 | - | 4 | 5 | 11 |

na, not applicable; ND, not determined
