# Supplementary material for: Nasopharyngeal carriage of Streptococcus pneumoniae, Haemophilus influenzae, and Staphylococcus aureus in a Brazilian elderly cohort
Source: PLoS One. 2019 Aug 22;14(8):e0221525. doi: 10.1371/journal.pone.0221525 (PMC6705818; doi:10.1371/journal.pone.0221525)
Supplement: S2 Table — (DOCX) [file pone.0221525.s002.docx]

**S2 Table.** Carrier status considering the subset of participants seen in both visit 1 and the follow-up visit (visit 2; n=584).

| Visit 1 | n | Follow-up Visit (visit 2) | | | | | | | | | | |  |
| --- | --- | --- | --- | --- | --- | --- | --- | --- | --- | --- | --- | --- | --- |
|  |  |  | *S. aureus* | |  | MRSA | |  | *S. pneumoniae* | | *H. influenzae* | | |
|  |  |  | positive | negative |  | positive | negative |  | positive | negative | positive | negative | |
|  | 584 |  | 105 | 479 |  | 18 | 566 |  | 15 | 569 | 10 | 574 | |
| *S. aureus* |  |  |  |  |  |  |  |  |  |  |  |  | |
| positive | 91 |  | 53 | 38 |  | 9 | 82 |  | 6 | 85 | 1 | 90 | |
| negative | 493 |  | 52 | 441 |  | 9 | 484 |  | 9 | 484 | 9 | 484 | |
| MRSA |  |  |  |  |  |  |  |  |  |  |  |  | |
| positive | 12 |  | 6 | 6 |  | 6 | 6 |  | 1 | 11 | 0 | 12 | |
| negative | 572 |  | 99 | 473 |  | 12 | 560 |  | 14 | 558 | 10 | 562 | |
| *S. pneumoniae* |  |  |  |  |  |  |  |  |  |  |  |  | |
| Positive | 9 |  | 1 | 8 |  | 0 | 9 |  | 1 | 8 | 1 | 8 | |
| negative | 575 |  | 104 | 471 |  | 18 | 557 |  | 14 | 561 | 9 | 566 | |
| *H. influenzae* |  |  |  |  |  |  |  |  |  |  |  |  | |
| positive | 14 |  | 3 | 11 |  | 2 | 12 |  | 2 | 12 | 1 | 13 | |
| negative | 570 |  | 102 | 468 |  | 16 | 554 |  | 13 | 557 | 9 | 561 | |
